# Interstate disparities in the performances in combatting COVID-19 in India: efficiency estimates across states

**DOI:** 10.1186/s12889-020-10051-6

**Published:** 2020-12-29

**Authors:** Shrabanti Maity, Nandini Ghosh, Ummey Rummana Barlaskar

**Affiliations:** 1 Department of Economics, Vidyasagar University, Paschim Midnapore, Midapore, West Bengal India; 2 Department of Microbiology, Vidyasagar University, Midnapore, West Bengal India; 3 Department of Economics, Assam University, Silchar, Assam India

**Keywords:** COVID-19, Stochastic production frontier, Inefficiency effects, Recovery rate

## Abstract

**Background:**

Currently, the novel coronavirus or COVID-19 pandemic poses the greatest global health threat worldwide, and India is no exception. As an overpopulated developing country, it is very difficult to maintain social distancing to restrict the spread of the disease in India. Under these circumstances, it is necessary to examine India’s interstate performances to combat COVID-19.

This study aims to explore twin objectives: to investigate the comparative efficiency of Indian states to combat COVID-19 and to unfold the factors responsible for interstate disparities in the efficiency in combatting COVID-19.

**Methods:**

The stochastic production frontier model was utilized for data analysis. The empirical analysis was facilitated by the inefficiency effects model, revealing the factors that influence interstate variability in disease management efficiency. Three types of variables, namely, output, inputs, and exogenous, were used to measure health system efficiency. The relevant variables were compiled from secondary sources. The recovery rate from COVID-19 was the output variable and health infrastructures were considered as the input variable. On the contrary, the non-health determinants considered to have a strong influence on the efficiency of states’ disease management, but could not be considered as input variables, were recognised as exogenous variables. These exogenous variables were specifically used for the inefficiency analysis.

**Results:**

The empirical results demonstrated the existence of disparities across Indian states in the level of efficiency in combatting COVID-19. A non-trivial outcome of this study was that Tamil Nadu was the best performer and Manipur was the worst performer of the investigated states. Variables such as elderly people, sex ratio, literacy rate, population density, influenced the efficiency of states, and thus, affected the recovery rate.

**Conclusion:**

This study argues for the efficient utilisation of the existing health infrastructures in India. Simultaneously, the study suggests improving the health infrastructure to achieve a long-run benefit*.*

**Supplementary Information:**

The online version contains supplementary material available at 10.1186/s12889-020-10051-6.

## Background

Millions of people worldwide are affected by severe acute respiratory syndrome coronavirus 2 (SARS-CoV-2), caused by the disease commonly known as COVID-19, rendering the disease a pandemic within a few months of the first case of infection. Combatting COVID-19 is a major challenge to all countries especially in the current situation with no vaccine or medicine for the treatment of the disease. Moreover, COVID-19is highly contagious, making it capable of rapidly spreading across the world. The World Health Organization (WHO) declared the disease a public health emergency of international concern [34]. Thus, countries have a very limited time to prepare for a battle against the unseen opposition. This fight more challenging for developing and underdeveloped countries with limited medical infrastructure, huge population pressure, backward economic conditions, amongst other problems. In this situation, containment, social distancing, and maintaining a healthy habit are criteria of the highest priority to confront the disease.

India is a developing country with a population of 136.64 crores, 2^nd^worldwide in terms of population size [29–31] and 19th in population density [29–31]. The World Bank [33] indicated that 24% of people live in densely populated slum areas in India (https://data.worldbank.org). Therefore, it is very difficult to maintain social distancing and stop the disease from spreading.

In India, the first case of COVID-19 infection was confirmed on 30th January 2020 in the state of Kerala. The complete lockdown was implemented in India on 24th March 2020, when only 564 people are infected by COVID-19 [4]; however, the number of infected persons continually increases and reached 1,97,945 on 25th May 2020. India is comprised of28 states and 8 union territories. The rate of infection and the recovery rates vary across different states. Several factors are also involved in the successful management of COVID-19 outbreaks in the states. Lu et al. [19], investigate the impact of quarantine on mental health. In this study, a comprehensive number of pertinent literature is reviewed to understand the outcomes of different dimensions of *COVID-19*. However, the specific issue of interstate disparities in the efficiency of controlling the *COVID-19* is not properly addressed, and it’s the first of its kind to address this issue. This backdrop motivates us to pursue this study. Accordingly, the objective of this study is twofold: the initial objective is *to investigate the comparative efficiency of different Indian states to combat COVID-19* and also attempt to unfold *the factors responsible for interstate divergences of efficiency in combatting COVID-19.*

From the concept of the neoclassical production function, efficiency is defined as an act of the economic agent to produce a specified output at minimum costs. This implies that the economic unit should choose inputs to minimise the production costs. However, concerning healthcare, the aftermath is the most important factor. Thus, health care efficiency fundamentally concerns the advancement of an individual’s health. This can be accomplished in two alternative ways, either maximum utilisation of current input level or enlargement of the available inputs to attain a higher outcome level. This can be implemented by identifying the health agents with better performance than others and exploring the constituents that help amplify their performances. Thus, a stochastic production frontier model for health economics could be specifically formulated. There exists pertinent literature concerning the application of stochastic production frontier model for assessing the performances of the health system. Studies which advocates the superiority of the stochastic production frontier approach for estimating the efficiency of health service providers, e.g., hospitals etc., are- Wagstaff [32], Hofler and Rungeling [15], Zuckerman et al. [36], Defelice and Bradford [9], Chirikos [5], Gerdtham et al. [13], Chirikos and Sear [6] and Street and Jacobs [28]. Moreover, studies by Murray and Frenk [22], Evans et al. [10], Sankar and Kathuria [25], Kathuria and Sankar [18], Forbes et al. [12], Hamidi [14] and Yildiz et al. [35], endorse that the ‘*Stochastic Frontier Approach (SFA)*’ is a robust approach to measure the efficiency of the health service.

This study is structured as follows. First, the *conceptual framework is discussed after the* introduction. In “Methodology” section, the data sources, variables, and the stochastic frontier model utilised for the empirical study are discussed. The empirical results and discussion are presented in “Results” section. Finally, “Conclusion” section concludes and draws policy implications.

## Methods

### Conceptual framework

**Fig. 1:**
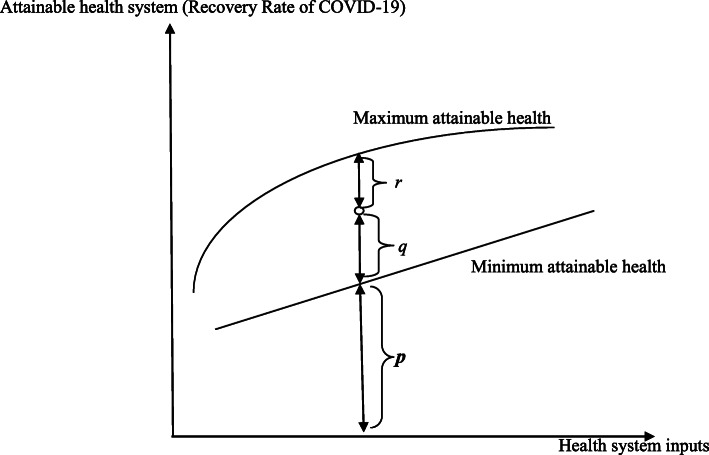
Health System Performance. Source: Adapted from Murray and Frenk [22] and Evans et al. [10]

In an attempt to recon the efficiency of different Indian states in combatting COVID-19, the notion of health system efficiency is elucidated following Murray and Frenk [22] and Evans et al. [10]. Following Murray and Frenk [22] and Evans et al. [10], the desired aim (*goal*) of the health system, in our case the ‘*recovery rate of COVID-19*’, was measured on the vertical axis as shown in Fig. 1.

The inputs to attain the desired outcome were measured on the horizontal axis. The upper line in the figure delineates the maximum possible health aftermath attainable from the given set of health inputs. In the literature, it is designated as ‘*frontier*’. The lower line in the figure portrays the level of attainable health sequelae in the absence of any health system. The principal contrast between the farm output and health system outcome is that in the absence of inputs, farm output would be zero. However, the health outcome would not be zero in the absence of any health expenditures, as all individuals in a nation will not die simultaneously.

We presumed that the country and/or the state had accomplished (*p + q*) units of health outcomes. The maximum possible attainable health outcome was *p + q + r* (see Fig. 1). Under this diegesis, ‘*system performance*’ is defined as [10, 22]:

1 $$ q/\left(q+r\right), $$

where (*q + r*) is the potential outcome and *q* is the level of health outcome achieved.

Thus, Eq. (1) can be interpreted as the ‘*system achieves compared to its potential*’ [22]. The question is how to measure the performance of the health system systematically; thus, we can permit inter -, intra -, and/ or state comparisons over time. This is thoroughly examined in this study in the context of combatting COVID-19.

In the ‘*frontier*’ framework, ‘*technical efficiency’ is* defined as the *‘farm’s capability to produce the maximum possible output from a given set of inputs’.* It is measured by the ratio of the observed to the maximum achievable outputs. In terms of Fig. 1 it, the *ratio, (p + q)/ (p + q + r)*. It is known as the *‘output-based measure of technical efficiency’* [20]. Thus, this definition was adopted to measure the performance of the health system of the states in combatting COVID-19 because in health system performance we are willing to measure the relationship between what the system attains relative to its potential. Thus, according to this definition, *health system efficiency was considered synonymous with health system performance*. In the subsequent discussion, the term ‘*efficiency*’ will be used to allude to ‘*system performance’*. In this study, we examined the measurement of technical efficiency only by using the ‘*stochastic frontier approach*’ (SFA) by considering availability and access of health care infrastructures as inputs and health sector performance as a single output. In this study, we examined the inefficiency effects of the stochastic production frontier Battese and Coelli [2]. Thus, the estimation followed a two-step procedure. First, the efficiency score of the different states of India was measured, and second, the components responsible for the differences in the performances of different Indian states in combatting COVID-19 were identified. The details specification of the econometric model is presented next.

## Methodology

This section discusses the data sources, the corresponding variables, and theoretical underpinning of the application of stochastic frontier analysis in health economics. This helps in formulating the stochastic production frontier (SPF) model to investigate the study objectives.

### Data

The study is entirely premised on secondary data compiled from various secondary sources. This study investigates 21 states and 1 Union Territory of India. The remaining Indian states and Union Territories are excluded due to the lack of available relevant data. The included states were Andhra Pradesh, Arunachal Pradesh, Assam, Bihar, Chhattisgarh, Gujarat, Haryana, Jharkhand, Karnataka, Kerala, Madhya Pradesh, Maharashtra, Manipur, Meghalaya, Orissa, Punjab, Rajasthan, Tamil Nadu, Uttar Pradesh, Uttarakhand and West Bengal and the union territory was Delhi. The study examined 20 independent variables, nine of which were input variables and 11 were exogenous variables. The variables were collected from various secondary sources. Table 1 provides a comprehensive summary of the variables and their sources.

**Table 1:** Descriptions of the variables [11, 16, 17, 23, 24]

Variables	Definition	Category	Data source
Recovery Rate (RR) *(y)*	Shows the ratio of total number of persons recovered from *Covid-19* to total number confirmed cases. The variable is calculated by the authors based on total number of persons recovered from *Covid-19* and total number confirmed cases.	Output	Ministry of Health and Family Welfare, GOI. Retrieve from: https://www.mohfw.gov.in/
Doctor-population ratio per 1000 (DOCTOR) *(x*_*1*_*)*	Shows the ratio of number of government allopathic doctors and population served in India per 1000 population. The variable is calculated by the authors based on the number of government allopathic doctors and population served in India. Then the ratio is transferred for per 1000 population.	Input	Directorate of State Health Services & National Health Profile. Retrieve from: http://www.cbhidghs.nic.in/
Nurses- population ratio per 1000 population (NURSE) *(x*_*2*_*)*	Shows the total number nurses served per 1000 population.	Input	Indian Nursing Council. Retrieve from: http://www.indiannursingcouncil.org/
Total Police per lakh of population (Police) *(x*_*3*_*)*	Shows number of police person served per lakh population.	Input	Ministry of Home Affairs. Retrieve from: https://www.mha.gov.in/
Num Isolation Beds (Isolation beds) *(x*_*4*_*)*	Total number of isolation beds available for COVID-19 patient.	Input	Retrieve from: https://www.covid19india.org/
Total People In Quarantine (Quarantine) *(x*_*5*_*)*	Total number of people kept in quarantine for observation.	Input	Retrieve from: https://www.covid19india.org/
Number of ICU beds (ICU beds) *(x*_*6*_*)*	The total number of intensive care unit beds available for the care of Covid-19 affected patients	Input	COVID-19 modelling estimates for India by a team of researchers affiliated with CDDEP and Princeton University, https://cddep.org/covid-19/hospital-capacity-in-india/india/
Number of ventilators (Ventilators) *(x*_*7*_*)*	The total number of ventilators available for the aiding artificial respiration during severe respiratory distress of Covid-19 affected patients	Input	COVID-19 modelling estimates for India by a team of researchers affiliated with CDDEP and Princeton University, https://cddep.org/covid-19/hospital-capacity-in-india/india/
Number of COVID testing labs (Labs) *(x*_*8*_*)*	Total number of laboratories for testing patient bio-fluid for Covid-19	Input	Indian Council of Medical Research (ICMR) report, 2020. Retrieve from: https://main.icmr.nic.in/
Sum of Total Tested (Tested) *(x*_*9*_*)*	Shows the total number of person actually tested for *covid-19.*	Input	Retrieve from: https://www.covid19india.org
Percentage of 60 plus (Elderly) *(z*_*1*_*)*	Refers the percentage share of 60 and above population in the total population.	Exogenous	Census of India, 2011 (For reference check reference list in the manuscript).
Sex Ratio (SR) *(z*_*2*_*)*	Shows the number of female population per 1000 male population.	Exogenous	Census of India, 2011
Literacy Rate (LR) *(z*_*3*_*)*	Defined as total number of literate persons in a given age group, expressed as a percentage of the total population in that age group	Exogenous	Census of India, 2011
Urbanisation (%) (Urban) *(z*_*4*_*)*	Shows the percentage share of population live in urban areas.	Exogenous	Census of India, 2011
Number of persons per room used for sleeping (Sleep) *(z*_*5*_*)*	Average number of persons using a single room for sleeping	Exogenous	National Family Health Survey-4, 2015–16, (For reference check reference list in the manuscript).
Percentage of self reported diabetes between age 15–49 (Diabetes) *(z*_*6*_*)*	The percentage of people between 15 and 49 yrs. of age in a population who have reported that they are suffering from diabetes	Exogenous	National Family Health Survey
Percentage of self reported heart disease between age 15–49 (Heart) *(z*_*7*_*)*	The percentage of people between 15 and 49 yrs. of age in a population who have reported that they are suffering from heart disease	Exogenous	National Family Health Survey
Population density/km2 (Population density) *(z*_*8*_*)*	Number of people living per square kilometre area	Exogenous	Census of India, 2011
Per capita NSDP (PCNSDP) *(z*_*9*_*)*	Net State Domestic Product (NSDP) is defined as a measure, in monetary terms, of the volume of all goods and services produced within the boundaries of the State during a given period of time after deducting depreciation, divided by total number of population.	Exogenous	Handbook of Statistics on Indian States, Reserve Bank of India, (For reference check reference list in the manuscript)
Regular wage/Salaried Employee (%) (Employment) *(z*_*10*_*)*	Shows the percentage share of workers engaged in regular salaried employment.	Exogenous	NSS, 68th Round, 2011–12, (For reference check reference list in the manuscript).
Internet subscriptions (per lakh population.) (Digitalisation) *(z*_*11*_*)*	Shows rate of internet subscription per lakh population.	Exogenous	Telecom Regulatory Authority of India. Retrieve from: https://www.trai.gov.in/

Source: Authors’ own specification

Table 1 shows the *recovery rate* from COVID-19, the output variable which was calculated as the percentage of recovery from COVID-19 and the total confirmed cases up to 25th May 2020.

### Variables

Three types of variables are indispensable for measuring health system efficiency using the *“stochastic production frontier model”* [10, 18, 25]. First, it is imperative to pinpoint a pertinent output indicator that represents the performance of the health sector. Second, it is mandatory to recognise a pertinent set of inputs that have an influence on the production of the output. Finally, it is highly recommended that variables that can affect the outcome of the health sector positively or negatively, but cannot be recognised as the inputs for the concerned output, are also included. These variables are non-health variables, categorised as ‘*exogenous’* variables. These exogenous variables seize the effects of non-health variables on health outcomes.

#### Output variable

Under *pandemic* circumstances, ensuring its citizens are safe and healthy is the most significant concern of the central and state government of India. India is a highly populated country with a population density of 1202 people per mi^2^. Under such circumstances*, COVID-19* may lead to catastrophic outcomes for India. In this, we consider *‘the rate of recovery’* from COVID-19 in different states of India as the output variable.

#### Input variables

Apropos input variables have two alternatives: either to use the monetary expenditures on health (such as per-capita expenditures on public health) or physical inputs. Due to the lack of data available, we decided to consider physical health inputs, which refer to the numbers of health care professionals, police, hospital facilities, etc. (Table 1). It is noteworthy that as population and area fluctuate across states, the variations in population size affect the access to available health facilities. Therefore, the ratios of different parameters with population size were calculated to obtain a clear picture of the available input variables per capita.

#### Exogenous variables

It is a well-established fact that improved health is not an exclusive outcome of the health service providers [22]. This is also true for combatting COVID-19. Some pioneering studies highlighted the influence of non-health determinants, such as income and educational level, measured differently [3, 26]. In this study, we investigated several variables that have a significant influence on the efficient control of COVID-19 across different states of India. However, these variables could not be categorised as input variables, and so these variables were categorised as *exogenous* variables. These variables were exclusively considered for the analysis of inefficiency effects and are listed in Table 1. It is worth noting that all the variables, viz., output, inputs and exogenous are representing the total figures of the Indian states, including both rural and urban statistics.

### Econometric model

Fundamentally, a stochastic frontier production function for the cross-sectional data can be manifested as follows:

2 $$ {y}_i=f\left({x}_i,\beta \right)\exp \left({V}_i\right)T{E}_i $$

Where, y is the health outcome, *x* and *β* stand for the vector of arguments of the production function, viz., access *and availability of the health infrastructure inputs, which are directly influencing the health outcomes* and the vector of the coefficients respectively; all the variables being expressed in logarithm. exp(*V*_*i*_) is the random error term and the subscript *i* refers to the particular cross-section, viz., the *ith* state.

The firm-specific technical efficiency Battese and Coelli [2] which is assumed to be random variable may be written as: *TE*_*i*_ = exp(−*U*_*i*_). Since *TE*_*i*_ ≤ 1, hence *U*_*i*_ ≥ 0, i.e., this error is one-sided. So, we can write (2) as:

3 $$ {y}_i=f\left({x}_i,\beta \right)\exp \left({V}_i\right)\exp \left(-{U}_i\right) $$

Here the assumptions are that ${V}_i\sim IIDN\left(0,{\sigma}_V^2\right)$ and ${U}_i\sim IIDN\left({z}_i\delta, {\sigma}_U^2\right)$.

Where *z*_*i*_ a (*1xm*) vector of explanatory variables is associated with the technical inefficiency of production across states and *δ* is an (*mx1*) vector of unknown coefficients.

Further *U*_*i*_ and *V*_*i*_ are independent of each other and also independent of *x*_*i*_. So, the underlying model is Normal-Truncated Normal [27].

It is noteworthy that the component *V*_*i*_ captures random variation in output due to factors outside the control of the state (suchlike, non-reporting of COVID-19 cases, etc.), which is uncontrollable and can be considered as the “*act of God*”. On the contrary, *U*_*i*_ reflects technical inefficiency on the part of the state and it is controllable. The technical efficiency [1] of the health sector for the *ith* state, *TE*_*i*_, in the stochastic frontier model (3) could be specified in Eq. (4).

4 $$ T{E}_i=\exp \left(-{U}_i\right)=\exp \left(-{z}_i\delta -{W}_i\right) $$

The random variable, *W*_*i*_ is defined by the truncation of the normal distribution with zero mean and variance ${\sigma}_U^2$, such that the point of truncation is −*z*_*i*_*δ*, that is, *W*_*i*_ ≥  − *z*_*i*_*δ*. These assumptions are consistent with *U*_*i*_ being a non-negative truncation of the $N\left({z}_i\delta, {\sigma}_U^2\right)$ distribution.

The *maximum likelihood estimation technique is the best way to estimate simultaneously the parameters of the stochastic frontier and the technical inefficiency model* [2]. *The likelihood function is expressed in terms of the variance parameters* [1], viz.,

${\sigma}^2={\sigma}_V^2+{\sigma}_U^2$and $\gamma =\frac{\sigma_U^2}{\sigma^2}$, where *γ* lies between 0 and 1 depending on the dominance of *σ* and *σ*_*u*_ respectively. Maximum-likelihood estimations (MLE) of equations and the corresponding efficiency scores of the listed states are obtained by using the FRONTIER-4.1 programme [7].

Accordingly, following Battese and Coelli [2], the model is estimated in terms of the following equation:

5 $$ {\displaystyle \begin{array}{l}\ln \left(R{R}_i\right)={\alpha}_0+{\alpha}_{DOCTOR}\ln (DOCTOR)+{\alpha}_{NURSE}\ln (NURSE)+{\alpha}_{Police}\ln (Police)+\\ {}{\alpha}_{Isolation\ beds}\ln \left( Isolation\ beds\right)+{\alpha}_{Quarantine}\ln (Quarantine)+{\alpha}_{ICU\  beds}\ln \left( ICU\  beds\right)+\\ {}{\alpha}_{Ventilators}\ln (Ventilators)+{\alpha}_{Labs}\ln (Labs)+{\alpha}_{Tested}\ln (Tested)+\left({V}_i-{U}_i\right)\end{array}} $$

Where, *ln* is the natural logarithm (i.e., to the base *e*), *α*_0_ is the *intercept term* and *α*_*j*_ s are the vector of the coefficients measuring the effects of the corresponding vector of arguments of the production function, viz., access *and availability of the health infrastructure inputs, which are directly influencing the health outcomes*.

The technical inefficiency effects were presumed to be defined by the following equation:

6 $$ {\displaystyle \begin{array}{l}{U}_i={\delta}_0+{\delta}_{Elderly}\ln (Elderly)+{\delta}_{SR}\ln (SR)+{\delta}_{LR}\ln (LR)+{\delta}_{Urban}\ln (Urban)+{\delta}_{Sleep}\ln (Sleep)+\\ {}{\delta}_{Diabetes}\ln (Diabetes)+{\delta}_{Heart}\ln (Heart)+{\delta}_{Population\ density}\ln \left( Population\ density\right)+{\delta}_{PCNSDP}\ln (PCNSDP)+\\ {}{\delta}_{Employment}\ln (Employment)+{\delta}_{Digitalisation}\ln (Digitalisation)+{W}_i\end{array}} $$

The vectors *δ* s apprehend the effects of the technical inefficiency of production across states.

Equations (5) and (6) are estimated using FRONTIER 4.1 [7].

## Results

The empirical results of our estimated econometrics and others are discussed in the following.

### State-wise recovery rate from COVID-19 in India

**Fig. 2:**
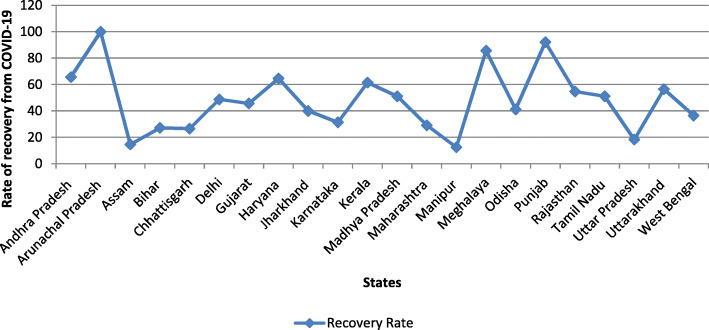
Interstate recovery rate from COVID-19. Source: Authors’ own graphical presentation

The COVID-19 pandemic had a very high metamorphosis and rate of infection. Although India is a densely populated country, prominent steps of the Indian government have limited the COVID-19 death toll to approximately 5 % of total infected cases. Considering the country’s immense population, the death rate is under control, although even a single death from COVID-19 would cause distress. The recovery rate of COVID-19 patients varied across the states. Figure 2 presents an overview of the state-wise recovery rate of COVID-19 in India.

Examination of the figure shows that the states’ recovery rates range from 100% (Arunachal Pradesh) to 12.5% (Manipur). The highest *recovery rate* from COVID-19 was identified in Arunachal Pradesh, followed by Punjab (92.14%). On the contrary, the lowest rate was identified in Manipur, followed by another north-eastern state, Assam (14.55%). The union territory Delhi was 11^th^in recovery rate with 48.74%. The trends of the two north-eastern states, Arunachal Pradesh and Assam, are noteworthy. These discrepancies in *recovery rate* may be attributable to the differences in the *efficiency* of existing health infrastructure utilisation amongst the states for combatting COVID-19. Furthermore, states with equal efficiency in combatting COVID-19 that had different recovery rates may be explained by *exogenous* factors influencing the state’s efficiency. In the subsequent sections, we discuss the variables that affect the efficiency of the states in combatting COVID-19.

### Efficiency analysis of different states in India

This section explores the main objective of this study, namely, the comparison of India’s interstate disparities in the efficiency in combatting *COVID-19*. However, we must first check the heterogeneity in the data. Descriptive statistics were imperative to understand the heterogeneity in the data, which is a paramount condition for any cross-sectional study. The descriptive statistics presented in Additional file 1: Table S1 confirm the heterogeneity in the data. The efficiency score and the corresponding ranking of the states based on the efficiency score are presented in Table 2. It is noteworthy that the corresponding efficiency score of the Indian states in conbatting COVID-19 are obtained by using the FRONTIER-4.1 programme developed by Coelli [7]. Significantly, relative efficiency scores exhibited how efficiently the states were performing in combatting COVID-19 in comparison to the most efficient state. The analysis of the relative health sector efficiency in combatting COVID-19 of the states was performed by considering the overall mean efficiency score of 0.709 as the benchmark of efficiency [8, 21]. It is worth noting that the corresponding value of S.D. is 0.232. The small value of S.D. also vindicates the use of mean efficiency score as the benchmark of efficiency for the comparison across Indian states. Consequently, a state would be categorised as relatively technically more efficient than other states if the achieved concerned state’s efficiency score was higher than mean efficiency and vice-versa. For example, West Bengal’s efficiency score was 0.932 higher than the overall mean efficiency score of 0.709. Thus, West Bengal was considered as a technically efficient state in combatting COVID-19 relative to other Indian states [8, 21]. Applying this benchmark, 14 of 22 Indian states were identified to be performing amply in combatting COVID-19, meaning 64% of Indian states were performing satisfactorily to combat COVID-19.

**Table 2:** Efficiency estimates and ranking for different states of India

States	Efficiency score	Ranking
Andhra Pradesh	0.892	5
Arunachal Pradesh	0.697	16
Assam	0.217	21
Bihar	0.735	11
Chhattisgarh	0.335	20
Delhi	0.938	2
Gujarat	0.885	6
Haryana	0.925	4
Jharkhand	0.801	9
Karnataka	0.877	8
Kerala	0.881	7
Madhya Pradesh	0.734	12
Maharashtra	0.577	18
Manipur	0.078	22
Meghalaya	0.706	15
Orissa	0.724	14
Punjab	0.796	10
Rajasthan	0.733	13
Tamil Nadu	0.949	1
Uttar Pradesh	0.659	17
Uttarakhand	0.525	19
West Bengal	0.932	3
Mean efficiency	0.709	**–**
Median efficiency	0.734	–
Std. Dev.	0.232	–

Source: Authors’ own calculation based on secondary data

The same table revealed the ranking of the Indian states based on efficiency scores, and the highest-ranking state was Tamil Nadu (0.949), followed by Delhi (0.938), and West Bengal (0.932).

### Analysis of stochastic frontier: factors affecting efficiency

This section focuses on dissecting the results of the stochastic production frontier estimation. The estimation of Eqs. 2 and 3 delineated the results of the stochastic production frontier and the inefficiency effects, respectively. The SPF, as presented in Eq. 2, could be considered as the log-linear version of the Cobb-Douglas production function. Maximum likelihood estimates of the parameters were obtained using the computer program FRONTIER 4.1 [7]. These estimates, together with the t-ratio, given to three significant digits, are presented in Table 3 and also in terms of the following equations:

**Table 3:** Maximum likelihood estimates of the stochastic production frontier function of performances in combating COVID-19 of different states of India (Dependent variable: LRR (Log of Recovery Rate) No of Observations: 20)

Variables	Coefficients		S.E	t-ratio
*Constant*	*β*_0_	0.109	0.093	1.165
*ln (DOCTOR)(x*_*1*_*)*	*β*_1_	0.151^a^	0.046	3.270
*ln (NURSE)(x*_*2*_*)*	*β*_2_	0.198^c^	0.113	1.754
*ln (Police)(x*_*3*_*)*	*β*_3_	0.082^c^	0.050	1.656
*ln (Isolation beds)(x*_*4*_*)*	*β*_4_	0.127^a^	0.048	2.624
*ln (Quarantine)(x*_*5*_*)*	*β*_5_	0.007	0.026	0.288
*ln (ICU beds)(x*_*6*_*)*	*β*_6_	0.273^b^	0.136	2.003
*ln (Ventilators)(x*_*7*_*)*	*β*_7_	−0.400^a^	0.080	−4.992
*ln (Labs)(x*_*8*_*)*	*β*_8_	3.410^b^	1.366	2.496
*ln (Tested)(x*_*9*_*)*	*β*_9_	−0.343^b^	0.166	− 2.074
*ln (Elderly)(z*_*1*_*)*	*δ*_1_	0.952^a^	0.302	3.150
*ln (SR)(z*_*2*_*)*	*δ*_2_	1.018^a^	0.388	2.621
*ln (LR)(z*_*3*_*)*	*δ*_3_	−3.078^a^	0.955	−3.223
*ln (Urban)(z*_*4*_*)*	*δ*_4_	−1.689^b^	0.825	− 2.048
*ln (Sleep) (z*_*5*_*)*	*δ*_5_	0.050	0.997	0.050
*ln (Diabetes) (z*_*6*_*)*	*δ*_6_	2.526^a^	0.703	3.591
*ln (Heart) (z*_*7*_*)*	*δ*_7_	1.829^b^	0.885	2.067
*ln (Population density) (z*_*8*_*)*	*δ*_8_	1.212^b^	0.589	2.059
*ln (PCNSDP) (z*_*9*_*)*	*δ*_9_	−0.668^b^	0.267	−2.506
*ln (Employment) (z*_*10*_*)*	*δ*_10_	1.610^a^	0.503	3.201
*ln (Digitalisation) (z*_*11*_*)*	*δ*_11_	−0.561	0.375	− 1.497
${\hat{\sigma}}_s^2$		1.689^b^	0.825	2.048
*γ*		0.265^c^	0.149	1.783
*μ*		0.920^a^	0.073	12.672
***Log (likelihood)***		−6.469		
***LR test***		16.445	Pr*ob* > *χ*^2^= 0.004	

Source: Authors’ own calculation based on secondary data

^a^significant at 1% level

^b^significant at 5% level

^c^significant at 10% level

The estimated regression equations are as follows:

***Stochastic Frontier*:**

7 $$ {\displaystyle \begin{array}{l}\ln \left(R{R}_i\right)=0.109+0.151\ln (DOCTOR)+0.198\ln (NURSE)+0.082\ln (Police)+\\ {}\kern4em (1.165)\kern1em (3.270)\kern6em (1.754)\kern5em (1.656)\\ {}0.127\ln \left( Isolation\ beds\right)+0.007\ln (Quarantine)+0.273\ln \left( ICU\  beds\right)\\ {}(2.624)\kern7.24em (0.288)\kern6.24em (2.003)\\ {}-0.400\ln (Ventilators)+3.410\ln (Labs)-0.343\ln (Tested)+\left({V}_i-{U}_i\right)\\ {}\left(-4.992\right)\kern6em (2.496)\kern4em \left(-2.074\right)\end{array}} $$

***Inefficiency Model:***

8 $$ {\displaystyle \begin{array}{l}{U}_i=0.920+0.952\ln (Elderly)+1.018\ln (SR)-3.078\ln (LR)-1.689\ln (Urban)+0.050\ln (Sleep)\\ {}\kern1.24em (12.672)\;(3.150)\kern5em (2.621)\kern3em \left(-3.223\right)\kern2.36em \left(-2.048\right)\kern3.24em (0.050)\\ {}+2.526\ln (Diabetes)+1.829\ln (Heart)-1.212\ln \left( Population\ density\right)-0.668\ln (PCNSDP)+\\ {}\kern0.24em (3.591)\kern5.24em (2.067)\kern4.12em (2.059)\kern9.24em \left(-2.506\right)\\ {}1.610\ln (Employment)-0.561\ln (Digitalisation)\\ {}(3.201)\kern7em \left(-1.497\right)\end{array}} $$

***Variance Parameter:***

$$ {\displaystyle \begin{array}{l}\begin{array}{rrr}{\hat{\sigma}}_s^2=1.689& \gamma =0.265& \mu =0.920\\ {}(2.048)& (1.783)& (12.672)\end{array}\\ {} Log\ (likelihood)=-6.469\ \mathrm{and} LR\  test=16.445\ \mathrm{and}\Pr ob>{\chi}^2=0.004\end{array}} $$

The absence of ‘*multicollinearity’* was countenanced by the Additional file 1: Table S2. The Table 3 disclosed that the output variable of the SPF was *‘recovery rate*’. The empirical estimates of Table 3 corroborated that the coefficients of the healthcare infrastructure combatting COVID-19, namely, *ln (DOCTOR), ln (NURSE), ln (Police), ln (Isolation beds), ln (ICU beds), ln (Ventilators), ln (Labs), and ln (Tested)* had the correct sign and were also statistically significant. These variables were widely recognised inputs for convalescence from COVID-19. The input variables *ln (DOCTOR), ln (NURSE), ln (Police), ln (Isolation beds), ln (ICU beds) and ln (Labs)* positively influencing the *recovery rate* from *COVID-19*. On the contrary, the input variables *ln (Ventilators) and ln (Tested)* negatively affect the *recovery rate* from *COVID-19*.

In this study, the estimated coefficients in the inefficiency are of particular interest. The estimated coefficients in the inefficiency model are also presented in Table 3. There was a positive and significant correlation with the elderly. The positive and significant estimates of sex ratio, population density, and employment infer that the antagonistic sex ratio, dense population, and underneath (regular wage/salaried employee) percentage (confirmed from ‘Additional file 1: Table S1’) affected the recovery rate of COVID-19 sceptically. Furthermore, higher self-reported diabetes and heart patients lower the efficiency of the state in combating COVID-19.

On the contrary, the negative and significant estimates of literacy rate, urbanisation, per-capita National State Domestic Product (NSDP), and digitalisation implied that the states with higher literacy rates, a greater proportion of urban areas, and improved per-capita NSDP, and improved digitalisation tended to be less inefficient.

The estimate for the variance parameter, *γ*, is significantly different from “0”, indicating that the inefficiency effects are likely to be highly significant for Indian states in combating COVID-19. It is worth noting that by the “*Generalized Likelihood-Ratio*” test we test the null hypothesis that the *inefficiency effects are absent* from the model, that is,

$$ {H}_0:\gamma ={\delta}_0={\delta}_1=....={\delta}_{11}=0 $$

The corresponding test statistics has a mixed *χ*^2^ distribution and the corresponding probability advocates that the null hypothesis is strongly rejected. This validates the inefficiency effects of the stochastic production frontier [2] model.

## Discussion

COVID-19 is spreading since December 2019. Due to the very high contamination rate of the disease, it has become a global pandemic. In India, this disease is creating a pandemonium along with drastic destruction of public health as well as the whole economy. Under such circumstances, scrutiny of the state’s efficiency can significantly help understand the level of achievement. As India is a diverse country, inter-state disparities also exist at the level of achievements. Thus, this study endeavours to highlight this aspect using the stochastic production frontier model.

The ranking of the Indian states based on efficiency score divulges that the most efficient state in combatting COVID-19 is Tamil Nadu and the least efficient state in the list is Manipur. It is noteworthy that the efficiency ranks only indicate the relative performance of the states and do not indicate any hierarchy in actual health outcomes. For example, the 3rd position was occupied by West Bengal which had a relative efficiency score of 0.932. However, in terms of actual attainment, the state ranked 15th in the recovery rate from COVID-19 amongst the 22 states, with a *recovery rate of* 36.51%.The relative health system efficiency score of the state stipulated that *given its health investment*, the state had accomplished approximately 93% of its prospective in resisting the spread of COVID-19. If the state’s health system operated as efficiently as the most efficient state in the study, this rate could have been 95%. On the contrary, if the state’s health system was as inefficient as the least efficient state Manipur, the resisting ability of the state could have diminished to approximately 8%, resulting in only a 12.5% recovery rate from COVID-19. This could be due to inappropriate utilisation of the available health infrastructure, leading to variations in the efficiency among different Indian states. This is the reason for the low level of health outcomes and achievements.

The absence of similar studies at the national and/or international level did not allow for cross verification of the obtained results in our study.

The empirical estimates of SFA model disclose the positive impact of the conventional health infrastructural inputs, such as *ln (DOCTOR), ln (NURSE), ln (Police), ln (Isolation beds), ln (ICU beds), and ln (Labs)* inferred that the increase in these inputs would improve the *recovery rate* from COVID-19. On the contrary, the negative sign of the estimated coefficients *ln (Ventilators)* and *ln (Tested)* due to the utilisation of ventilators for serious patients and expansion of proper COVID-19 testing enabled us to identify appropriate COVID-19 affected cases. Consequently, the number of confirmed COVID-19 cases increased. The expansion of ventilators utilisation indicated an increase in serious COVID-19 cases. Simultaneously, the augmentation of proper COVID-19 tests will accelerate the number of confirmed COVID-19 cases. The cumulative effects of these two conventional health infrastructural inputs may gradually reduce the recovery rate from COVID-19. It is noteworthy that, in the prevailing circumstances, *ln (Police)* emerges as a predominant input variable. The positive and significant influence of the estimated coefficient re-establishes the patent fact. The positive and significant footprints of the estimated coefficient *ln (Isolation beds) and ln (ICU beds)* were also expected. Isolating the COVID-19 patient from others through isolation beds and ameliorated medical equipment in the multi-speciality ICU would also increase the recovery rate from COVID-19.

Social, economic, and demographic variables were also included for analysis. However, these variables could not be considered input variables. Nevertheless, their influence on the efficiency of the state in combatting COVID-19 could not be disregarded. Thus, these variables were categorised as exogenous variables. These exogenous variables were considered as the drivers of efficiency. Variables such as elderly people, sex ratio, literacy ratio, population density, per capita NSDP, co-morbidity rate (here self-reporting of heart disease and diabetes), regular wage earners, influenced the efficiency of the states and consequently affected the recovery rate. The positive significant correlation with the exogenous variable elderly indicated that the state with a higher percentage of older adults was more inefficient than a state with a relatively *younger population*. Moreover, as older adults are vulnerable to COVID-19 with a lower survival rate, this consequently leads to a lower ‘*recovery rate*’. The results analogous to *sex ratio*, *population density*, and *employment* are quite apparent in the case of India, where the mean sex-ratio and population density/km^2^ were approximately 951 and 921, respectively. The results corresponding to the sex ratio are patently true. A population with equal and/or more proportion of the female population stipulates a favourable sex ratio. As women in Indian society are likely to stay at home, the greater female population enhances the potentiality of successful *‘lockdown’* without active coercion from the *police force*. It is a well-established fact that densely populated areas are vulnerable to community contamination of COVID-19*.* Thus, these two exogenous variables can predictably influence the inefficiency of the state to combat COVID-19. Furthermore, the mean (regular wage/salaried employee) percentage for Indian states was approximately 27%, indicating that a significant proportion of working adults were regular wage workers. Consequently, if they do not perform their duties, they are not eligible to receive salaries. These labour forces will certainly become a deterrent for implementing a successful lockdown. Lockdown was a globally accepted mechanism to restrict the community spread of COVID-19*.* For these workers, *livelihood is equally important as life*. In search of livelihood, these workers may unwillingly break the lockdown rule and unfortunately lead to the failure of the lockdown. The positive and significant estimates of self-reported diabetes and heart patients also reflect accurate facts. In fact, co-morbidity is one of the reasons for the poor recovery rate.

The negative and significant influence of the exogenous variables on *literacy rate*, urbanisation, *per-capita National State Domestic Product (NSDP)*, and *digitalisation* may be due to the following reason. A higher *literacy rate* means the population is more aware and has a greater probability of successful ‘*lockdown*’. Normally, urban areas are typified with modern facilities of health, law and order, social services facilities. Urban areas normally retain multispecialty hospitals with modern ICU, ventilators, a significant number of the police force and civic volunteers, social volunteers, fast internet services, and online availability of necessary goods, including medicines. A state with a greater proportion of urban areas is more efficient in combatting COVID-19. A high per-capita NSDP is a barometer of the state’s prosperity. States with higher per-capita NSDP may experience upgraded health infrastructure and improved human capital (i.e., healthy and educated citizens). Thus, the likelihood of the state combatting COVID-19 is greater. This is the reason for the positive sequel of the per capita NSDP on the efficiency of the state in combatting COVID-19.

The awareness about ‘*do’s and don’ts*’ related to COVID-19 is well managed through the internet. Transactions through ‘net banking’ and ‘online purchase of necessary goods’ become key constituents for ensuring a successful ‘*lockdown*’. Consequently, the states with improved internet facilities will experience successful “*Lockdown*” and consequently counter COVID-19 more efficiently than other states.

It is worth noting the empirical analyses of the present paper are based on the total statistics, including both rural and urban areas of Indian states. In the case of India rural urban demarcation, specifically concerning the availability of the health care resources (doctors, nurses, hospitals etc.), is very salient. The urban India is more equipped with all kinds of health care resources (doctors, nurses, hospitals etc.) and as expected to be more efficient to combat COVID-19. Consequently a separate analysis for rural and urban India is always better. Unfortunately, non-availability of the relevant statistics dictated us to consider total statistics (considering both rural and urban together) for the empirical analyses. However, we have considered “*Urbanisation*” as one of the exogenous variable to apprehend the role of “*Urbanisation*” in the efficiency in combating COVID-19. This is definitely a limitation of this study. Moreover, the ranking of the states remains invariant because the efficiency scores of the states are obtained by using the FRONTIER-4.1 programme.

## Conclusion

This study is predominantly empirical. The empirical results allow us to confirm the existence of *interstate disparities in the efficiency in combatting COVID-19 across India*. This was evident in the variation in the recovery rate across Indian states. Various social, economic, and demographic variables increased the efficiency of states in combatting COVID-19. Based on our empirical results, we suggest the following policy prescriptions:

First, as it is evident that the health infrastructure inputs, such as doctors, nurses, police, isolation beds, ICU beds, help the state escalate the recovery rate from COVID-19. Therefore, improvements in the health infrastructure will act as catalysts in fighting the pandemic and the health system as a whole in the long-term. Doctors, nurses, and police should be sufficiently provided with required medicines and relevant safety measures as they are frontline workers in this COVID-19 pandemic. Second, the exogenous variable, *the elderly population,* is a major component contributing to inefficiency. As they are more vulnerable to disease, utmost care should be provided to them along with required medical treatment and nutritious foods. Third, as population density may negatively affect efficiency, areas with dense populations may be considered for special surveillance to minimise contamination. Fourth, regular wage earners are in search of their livelihoods, and may unknowingly cause the lockdown to fail. These activities not only enhance the likelihood of their contamination but also community spread. To avoid such circumstances, it is highly recommended to arrange an alternative livelihood for them temporarily so that they stay at home and help in completing the lockdown successfully. Simultaneously, for salaried people, it is necessary to arrange *work from home* so that they can continue their duties to ensure the economy is running. Finally, in these days of hardship, the government of all states along with the centre should be very effectively controlling the damage, assuring the citizens’ safety and provision of requirements accordingly.

As mentioned earlier the empirical analyses of the present paper are based on the total statistics, including both rural and urban areas of Indian states. Depending on the availability of the data the paper can be extended considering rural and urban areas across Indian states in future.

## Supplementary Information

**Additional file 1: Table S1.** Descriptive statistics of output, input and exogenous variables. **Table S2.** Correlation Matrix.

## Data Availability

The study is based on the secondary data and all the data sources are clearly mentioned in the text. For further details kindly consult Table 1.

## Abbreviations

WHO: World Health Organization
UNO: United Nations Organisations
SFA: Stochastic frontier analysis
SPF: Stochastic production frontier
ICU: Intensive care unit
NSDP: National State Domestic Product
