# Supplementary material for: Interstate disparities in the performances in combatting COVID-19 in India: efficiency estimates across states
Source: BMC Public Health. 2020 Dec 29;20:1925. doi: 10.1186/s12889-020-10051-6 (PMC7770151; doi:10.1186/s12889-020-10051-6)
Supplement: Supplementary file 1 — Additional file 1: Table S1. Descriptive statistics of output, input and exogenous variables. Table S2. Correlation Matrix. [file 12889_2020_10051_MOESM1_ESM.docx]

**Additional file 1**

**Table S1: Descriptive statistics of output, input and exogenous variables**

| **Variables** | **Mean** | **Std. Dev.** | **Maximum** | **Minimum** | **Obs** |
| --- | --- | --- | --- | --- | --- |
| Recovery Rate | 47.94 | 23.93 | 12.5 | 100 | 22 |
| *ln(* Recovery Rate*)* | 3.74 | 0.56 | 2.53 | 4.61 | 22 |
| Doctor-population ratio per 1000 | 0.14 | 0.13 | 0.04 | 0.45 | 22 |
| Nurses per 1000 population | 2.42 | 1.87 | 0.18 | 7.61 | 22 |
| Total Police per lakh of population | 256.51 | 239.84 | 74.8 | 962.7 | 22 |
| Num Isolation Beds | 9205.68 | 13968.41 | 300 | 55707 | 22 |
| Total People In Quarantine | 7881.29 | 10900.65 | 1 | 38703 | 22 |
| No. of ICU bed | 3918.86 | 4186.01 | 90 | 14070 | 22 |
| No. of ventilators | 1982.09 | 2074.42 | 45 | 7035 | 22 |
| No. of COVID testing labs | 25.00 | 20.98 | 2 | 72 | 22 |
| SUM of Total Tested | 14897.55 | 14410.23 | 136 | 55678 | 22 |
| Percentage of 60 plus | 7.82 | 1.82 | 4.6 | 12.6 | 22 |
| Sex ratio | 951.27 | 47.67 | 868 | 1084 | 22 |
| Literacy rate % | 74.33 | 7.57 | 61.8 | 94 | 22 |
| Urbanisation % | 32.75 | 17.85 | 11.29 | 97.50 | 22 |
| No. of persons per room used for sleeping | 2.73 | 0.40 | 1.8 | 3.4 | 22 |
| Percentage of self reported diabetes between age 15-49 | 3.00 | 1.27 | 1.1 | 5.6 | 22 |
| Percentage of self reported heart disease between age 15-49 | 2.82 | 1.59 | 1 | 6.5 | 22 |
| Population density/km2 | 920.64 | 2336.40 | 17 | 11297 | 22 |
| Per capita NSDP | 107036.70 | 57660.97 | 30617 | 279601 | 22 |
| Regular wage/Salaried Employee (%) | 27.04 | 12.56 | 14.85 | 77.25 | 22 |
| Internet subscriptions (per lakh of population)(Digitalisation) | 20.37 | 12.86 | 0.85 | 37.67 | 22 |

*Source: Authors’ own calculation based on secondary data*

**Table S2: Correlation Matrix**

| **Variable** | x1 | x2 | x3 | x4 | x5 | x6 | x7 | x8 | x9 | z1 | z2 | z3 | z4 | z5 | z6 | z7 | z8 | z9 | z10 | z11 |
| --- | --- | --- | --- | --- | --- | --- | --- | --- | --- | --- | --- | --- | --- | --- | --- | --- | --- | --- | --- | --- |
| x1 | 1 |  |  |  |  |  |  |  |  |  |  |  |  |  |  |  |  |  |  |  |
| x2 | 0.34 | 1 |  |  |  |  |  |  |  |  |  |  |  |  |  |  |  |  |  |  |
| x3 | 0.43 | 0.22 | 1 |  |  |  |  |  |  |  |  |  |  |  |  |  |  |  |  |  |
| x4 | -0.39 | 0.17 | -0.29 | 1 |  |  |  |  |  |  |  |  |  |  |  |  |  |  |  |  |
| x5 | -0.34 | 0.04 | -0.44 | 0.42 | 1 |  |  |  |  |  |  |  |  |  |  |  |  |  |  |  |
| x6 | -0.40 | 0.25 | -0.44 | 0.42 | 0.45 | 1 |  |  |  |  |  |  |  |  |  |  |  |  |  |  |
| x7 | -0.50 | 0.19 | -0.39 | 0.50 | 0.42 | 0.51 | 1 |  |  |  |  |  |  |  |  |  |  |  |  |  |
| x8 | -0.41 | 0.25 | -0.40 | 0.45 | 0.49 | 0.40 | 0.49 | 1 |  |  |  |  |  |  |  |  |  |  |  |  |
| x9 | -0.48 | 0.24 | -0.42 | 0.46 | 0.51 | 0.48 | 0.48 | 0.47 | 1 |  |  |  |  |  |  |  |  |  |  |  |
| z1 | -0.33 | 0.42 | -0.38 | 0.47 | 0.37 | 0.50 | 0.41 | 0.45 | 0.45 | 1 |  |  |  |  |  |  |  |  |  |  |
| z2 | 0.07 | 0.25 | 0.21 | -0.16 | -0.36 | 0.05 | 0.00 | -0.03 | -0.18 | 0.34 | 1 |  |  |  |  |  |  |  |  |  |
| z3 | 0.39 | 0.43 | -0.03 | 0.11 | 0.20 | 0.33 | 0.30 | 0.29 | 0.11 | 0.44 | 0.26 | 1 |  |  |  |  |  |  |  |  |
| z4 | 0.32 | 0.42 | 0.03 | 0.35 | 0.20 | 0.41 | 0.40 | 0.47 | 0.34 | 0.43 | -0.12 | 0.44 | 1 |  |  |  |  |  |  |  |
| z5 | -0.42 | -0.34 | -0.42 | 0.30 | 0.39 | 0.40 | 0.42 | 0.43 | 0.40 | 0.09 | -0.54 | -0.29 | -0.02 | 1 |  |  |  |  |  |  |
| z6 | 0.34 | 0.35 | 0.22 | -0.01 | -0.06 | 0.00 | -0.02 | 0.04 | -0.17 | 0.20 | 0.34 | 0.39 | 0.20 | -0.43 | 1 |  |  |  |  |  |
| z7 | 0.05 | 0.20 | 0.16 | -0.10 | -0.10 | 0.03 | 0.00 | 0.12 | -0.11 | 0.21 | 0.65 | 0.14 | -0.04 | -0.45 | 0.48 | 1 |  |  |  |  |
| z8 | -0.06 | 0.07 | -0.47 | 0.27 | 0.46 | 0.42 | 0.51 | 0.47 | 0.45 | 0.43 | -0.24 | 0.46 | 0.48 | 0.29 | 0.24 | 0.03 | 1 |  |  |  |
| z9 | 0.35 | 0.41 | -0.02 | 0.26 | 0.06 | 0.42 | 0.47 | 0.41 | 0.32 | 0.38 | -0.09 | 0.48 | 0.43 | -0.01 | 0.10 | -0.10 | 0.39 | 1 |  |  |
| z10 | 0.48 | 0.36 | 0.04 | 0.12 | 0.07 | 0.30 | 0.23 | 0.31 | 0.18 | 0.03 | -0.28 | 0.45 | 0.42 | -0.12 | 0.19 | -0.03 | 0.50 | 0.44 | 1 |  |
| z11 | -0.49 | 0.07 | -0.46 | 0.44 | 0.40 | 0.45 | 0.42 | 0.44 | 0.48 | 0.44 | -0.12 | 0.10 | 0.29 | 0.46 | -0.22 | -0.19 | 0.43 | 0.31 | 0.11 | 1 |

*Source: Authors’ own calculation based on secondary data*
